# Potassium Dichromate Exposure and Sperm Quality in Rats: A Multi-Generational Study

**DOI:** 10.3390/toxics14100870

**Published:** 2026-09-30

**Authors:** Jelena Savici, Simona Marc, Oana-Maria Boldura, Shady Shalaby, Malina Oprescu, Diana Brezovan, Florin Muselin, Violeta Igna

**Affiliations:** 1 Faculty of Veterinary Medicine, University of Life Sciences “King Mihai I” from Timisoara, Calea Aradului No. 119, 300645 Timisoara, Romania; jelenasavici@usvt.ro (J.S.); malina.oprescu.iosud@usvt.ro (M.O.); dianabrezovan@usvt.ro (D.B.); florinmuselin@usvt.ro (F.M.); violetaigna@usvt.ro (V.I.); 2 Faculty of Veterinary Medicine, Mansoura University, El Gomhouria St., El Mansoura 35516, Egypt; shadyshalaby25@mans.edu.eg

**Keywords:** chromium, sperm, histopathology, rats, multi-generations

## Abstract

This study evaluated the effects of potassium dichromate on sperm quality and testicular histopathology across three generations of male rats. Animals received potassium dichromate in drinking water at 25, 50, or 75 mg/L, while controls received chromium-free water. Exposure was maintained across generations, and sperm quality and testicular histopathology were assessed. Cr(VI) exposure impaired sperm quality in a dose- and generation-dependent manner. The most pronounced effects occurred in the F2 generation at 75 mg/L, where sperm concentration decreased by approximately 31.35%, total motility by 66.79%, and progressive motility by 84.5% compared with controls. Morphological abnormalities increased from 9.86% in F2 controls to 63.43% in F2-E3 animals and involved the head, neck, and tail regions. Histopathological alterations also increased with dose and generation, with the testicular injury score increasing from 0.64 in F2 controls to 3.71 in F2-E3 animals, while the Johnsen score decreased from 9.65 in F2 controls to 6.54 in F2-E3 animals. Testicular lesions included seminiferous epithelium disorganisation, germ and Sertoli cell loss, germ cell detachment, Leydig cell degeneration, and defective spermiation. Overall, the greatest sperm and testicular alterations occurred in F2 animals, indicating cumulative multigenerational reproductive toxicity following Cr(VI) exposure.

## 1. Introduction

Accumulating evidence indicates a decline in male reproductive health over recent decades, particularly in sperm concentration and total sperm count, raising concerns about the contribution of environmental and lifestyle factors to male infertility [1,2]. In addition to genetic influences, lifestyle factors and environmental pollution have been implicated in impaired male reproductive function [1,3,4]. Environmental contaminants, including heavy metals released into aquatic environments, may adversely affect spermatogenesis and semen quality, thereby increasing the risk of male subfertility or infertility [5,6]. Spermatogenesis, the process essential for male fertility, involves a complex differentiation process consisting of spermatogonial mitosis, meiosis, and spermiogenesis [7].

These pollutants include endocrine disruptors, pesticides, and heavy metals [8]. Among heavy metals, chromium (Cr) and its compounds are widely used in industries such as electroplating, leather production, textiles, metallurgy, and pigments [9]. Wastewater and residues from Cr mining and metallurgical and chemical industries are major sources of soil and river contamination. Chromium exists mainly in two stable valence states: Cr(III) and Cr(VI). The latter is a major environmental pollutant [10] and is recognised as both an endocrine disruptor and a reproductive toxicant [11,12]. Industrialisation has sharply increased Cr(VI) levels in the environment [13]. Humans are exposed through contaminated soil, water, low-quality leather, occupational contact, cigarette smoke, and industrial emissions [14].

Cr(VI) enters cells via nonspecific anion channels, where its intracellular reduction to Cr(V), Cr(IV), and ultimately Cr(III) can generate reactive oxygen species (ROS), contributing to toxicity [15]. Cr(VI) exposure has been linked to disorders affecting multiple systems, including the respiratory [16] cardiovascular, digestive, renal, nervous [14], reproductive [17,18], as well as endocrine glands such as the pituitary [19,20], thyroid [21,22,23,24,25,26], pancreas [27,28,29,30], adrenal glands [31]. It also exhibits cytotoxic, genotoxic, immunotoxic, and carcinogenic effects in both humans and animals [32,33,34].

Exposure to Cr(VI) severely disrupts spermatogenesis and reduces sperm quality [35]. Studies have shown that Cr(VI) decreases sperm concentration and progressive motility in both animals and humans [36,37,38,39]. Epidemiological research further establishes a dose–response relationship between Cr(VI) levels in blood, sperm, and urine, and declining sperm quality [40,41,42]. Workers in industries like welding face a heightened risk of poor sperm quality, sperm abnormalities, and infertility, potentially leading to developmental issues in offspring [43]. Animal studies also indicate that Cr(VI) exposure increases sperm abnormalities while decreasing sperm concentration [36,37,44,45].

Evidence from previous research has shown that hexavalent chromium is capable of crossing the placental barrier, thereby posing a potential risk to offspring during intrauterine development [46]. In an independent study by our research group using different animals but an experimental protocol comparable to the present study, chromium exposure resulted in significant, dose-related accumulation in the reproductive organs and accessory sex glands of both the parental (F0) and F1 generations. Notably, chromium concentrations were consistently higher in the F1 generation than in the directly exposed F0 animals [47]. This progressive accumulation in the male reproductive organs was accompanied by significant endocrine disturbances, as demonstrated in our previous study [48]. Specifically, serum testosterone and luteinizing hormone (LH) levels showed an inverse pattern, with testosterone concentrations decreasing and LH concentrations increasing with both chromium exposure level and generation.

Despite evidence of Cr(VI)-induced reproductive toxicity in directly exposed animals and their offspring, information regarding the persistence of these effects across successive generations remains limited, particularly for the F2 generation. Specifically, the consequences of Cr(VI) exposure for testicular histology and sperm quality in F2 offspring remain insufficiently characterised. This knowledge gap, together with our previous findings, provided the rationale for the present investigation. Accordingly, the current study aimed to extend our understanding beyond hormonal alterations by evaluating the effects of chromium exposure on testicular histological architecture and sperm quality across three successive generations.

## 2. Materials and Methods

The study was conducted on three generations of rats. The F0 generation consisted of 28 male and 28 female, adult and healthy Wistar rats weighing 220–240 g. The animals were obtained from an authorised supplier, the University of Medicine and Pharmacy “Victor Babeș” Timisoara, and were acclimatised for one week under laboratory conditions, maintaining a temperature of 22 ± 2 °C, a relative humidity of 40–60%, and a 12 h light/12 h dark cycle. Males from the F1 and F2 generations were offspring produced during the experiment. The animals were housed in standard polycarbonate cages (750 × 720 × 360 mm) and maintained on a standard diet (Biovetimix code 140-501, Biovet, Tanacu, Romania) with access to food and, according to group allocation, either drinking water or potassium dichromate-containing drinking water throughout the experiment. The experimental protocol was approved by the Ethical Committee of Banat University of Agricultural Sciences and Veterinary Medicine “King Michael I of Romania” Timisoara (No. 60/23.07.2019).

The source of hexavalent chromium used in this study was obtained by diluting potassium dichromate (K_2_Cr_2_O_7_), a highly soluble chromium salt, in distilled water to obtain three concentrations: 25 mg/L, 50 mg/L, and 75 mg/L. Potassium dichromate (Sigma-Aldrich, Darmstadt, Germany) with analytical purity of at least 99%, was kept in well-sealed containers in a dry environment at room temperature, with protection from light and humidity to ensure its stability.

### 2.1. Experimental Design

**Figure 1:**
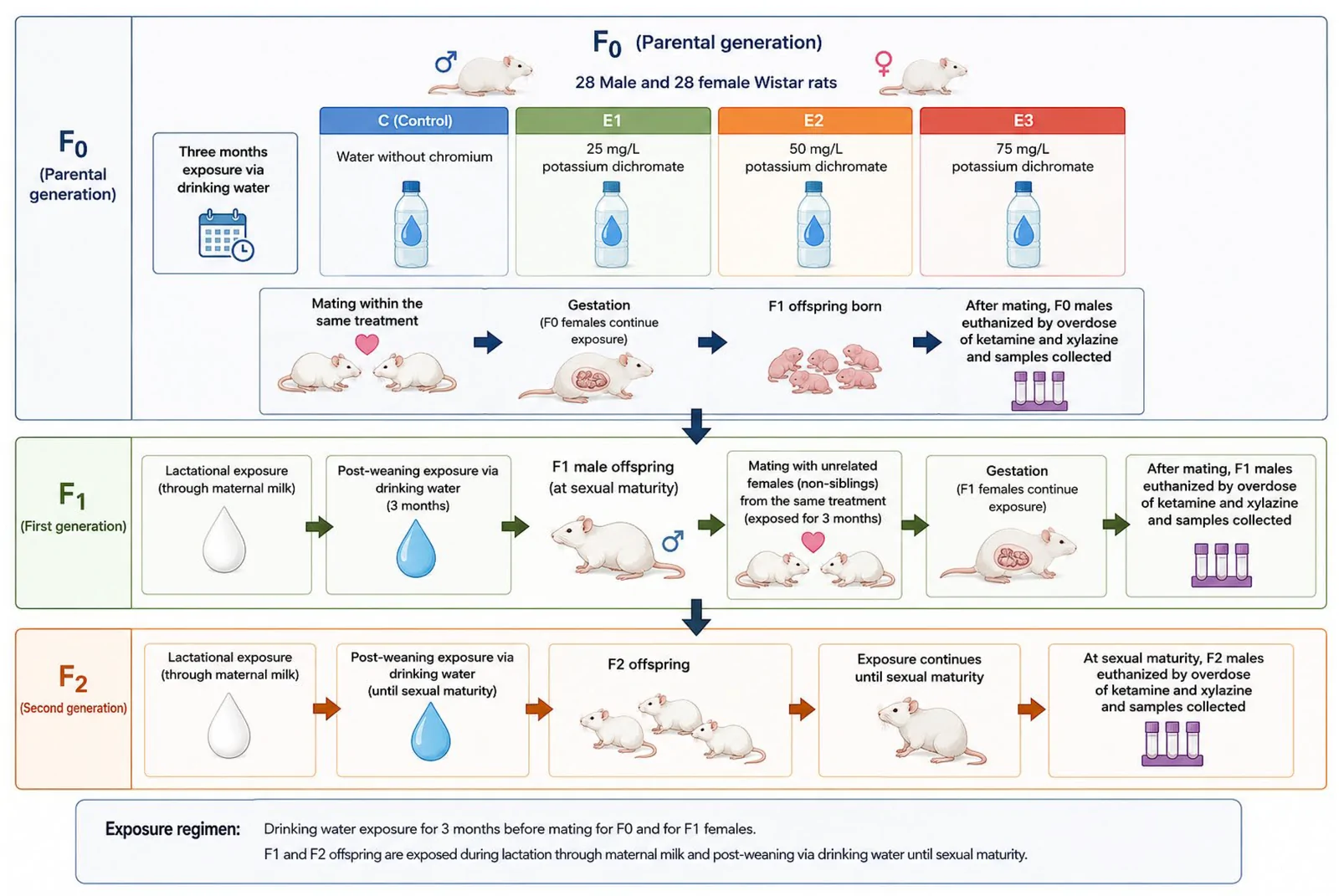
Three-generation mating experiment schedule (generated with AI).

The study was conducted in three stages (Figure 1).

Stage I—The male and female rats, representing F0 generation, were randomly assigned to one control (C) and three experimental (E) groups for each sex (n = 7 per group). Three months prior to mating, animals from experimental groups received potassium dichromate in drinking water:

- group E1—25 mg/L potassium dichromate,
- group E2—50 mg/L potassium dichromate, and
- group E3—75 mg/L potassium dichromate.

Rats from group C served as controls and received drinking water without potassium dichromate.

The K_2_Cr_2_O_7_ concentrations used in the present study (25, 50, and 75 mg/L) were selected to provide a graded exposure range, with 25 mg/L serving as the baseline concentration. Previous toxicological studies using comparable chromium exposures supported the selection of this baseline level. Acharya et al. [49] exposed male Wistar rats to 25 mg/L chromium administered as potassium dichromate in drinking water, while Sivakumar et al. [50] used 25 mg/L K_2_Cr_2_O_7_ in drinking water in an experimental model of reproductive toxicity. We included higher concentrations to assess whether the effects followed a concentration-dependent pattern.

After mating, all males were sacrificed by overdose of anaesthetic ketamine (Ketamine 10%, CP Pharma, Burgdorf, Germany) and xylazine (Narcoxyl, Intervet International, Boxmeer, The Netherlands). Sperm samples were collected from the vas deferens for sperm quality assessment, after which the testes were collected for histological examination.

Stage II—During gestation and lactation, F0 females continued to receive potassium dichromate in their drinking water at the same concentration as before mating. Following weaning, male offspring (F1 generation) were separated from female offspring and continued to receive potassium dichromate in their drinking water at the same concentration as their respective parental group for an additional three months, until reaching sexual maturity. At sexual maturity, we selected seven F1 males from each treatment group for reproductive evaluation. To ensure representation of the available litters within each treatment group, males were selected as equally as possible across litters, based on their body development. When fewer litters were available than males required for evaluation, we included more than one male from the same litter. The remaining male offspring were allocated to other experimental assessments that were outside the scope of the present study.

The seven selected F1 males from each treatment group were mated with unrelated (non-sibling) females exposed to the corresponding concentration of potassium dichromate in their drinking water for three months. Following mating, the F1 males were euthanised using the same protocol described for the F0 generation. The collected samples were analysed using the same procedures described for the F0 generation to assess sperm quality parameters and testicular histology.

Stage III—During gestation and lactation, F1 females continued to receive potassium dichromate in their drinking water at the same concentration as before mating. Following weaning, male offspring (F2 generation) were separated from female offspring and continued to receive potassium dichromate in their drinking water at the same concentration as their respective parental group for an additional three months, until reaching sexual maturity. At the end of the exposure period, seven F2 male rats from each treatment group were selected for evaluation using the same approach as described for the F1 generation. The selected F2 male rats were euthanised using the same protocol described for the F0 and F1 generations. We collected sperm and testicular samples and performed the same analyses of sperm quality parameters and testicular histology as for the F0 and F1 generations.

Animal handling during the experiment and their euthanasia were conducted in compliance with EU Directive 2010/63/EU [51] and NRC Guidelines [52].

### 2.2. Assessment of Reproductive Outcomes

Reproductive outcomes were assessed for each experimental group. Mating was performed using a 1:1 male-to-female ratio and was confirmed by the presence of a vaginal plug. The mating confirmation rate was calculated as the number of females with a confirmed vaginal plug divided by the total number of females paired for mating, multiplied by 100. Animals for which mating was not confirmed were excluded from the subsequent assessment of reproductive outcomes and were subsequently allocated to other investigations unrelated to the present study.

Gestation was assessed by abdominal palpation on day 12 after confirmed mating. The gestation rate was calculated as the number of females with confirmed gestation divided by the number of females with a confirmed vaginal plug, multiplied by 100. The parturition rate was calculated as the number of females that delivered offspring divided by the number of females with confirmed gestation, multiplied by 100.

The total number of pups, the number of live pups at birth, and the number of pups surviving to sexual maturity were expressed as mean ± standard deviation (SD). The postnatal survival rate was calculated for each experimental group as the total number of offspring surviving to sexual maturity divided by the total number of live-born offspring, multiplied by 100.

### 2.3. Sperm Assessment

The vas deferens were excised immediately after euthanasia. Spermatozoa were collected from both the right and left vas deferens by gently applying pressure along the entire length of each duct. The expressed sperm samples were collected into sterile microcentrifuge tubes, diluted in 300 μL pre-warmed Tyrode saline solution (Sigma-Aldrich Chemie GmbH, Steinheim, Germany) and processed immediately for subsequent sperm assessment.

Sperm motility and concentration were determined using a computer-assisted sperm analysis (CASA) system (IVOS, version 12.3, Hamilton Thorne Bioscience, Beverly, MA, USA) and Animal Motility Software with HTB IVOS parameter settings recommended for rat semen. Image acquisition was performed at a frame rate of 60 Hz, with 30 frames analysed per field. Measurements were performed in dark-field mode using a standard 10× objective, with the temperature maintained at 37 °C. For cell detection, the minimum contrast was set to 80 and the minimum cell size to 7 pixels, while the default settings for cell size, cell intensity, and cell contrast were 25, 80, and 15, respectively. Progressive spermatozoa were defined using a path velocity (VAP) threshold of 50.0 μm/s and a straightness (STR) threshold of 80.0%. Slow spermatozoa were classified as static using VAP and VSL cutoffs of 20.0 and 30.0 μm/s, respectively. Static intensity gates were set at 0.14–1.84, static size gates at 0.72–8.82, and static elongation gates at 0–47.

For each animal, 3 μL of the diluted sperm sample was loaded into one chamber of a Leja 4-chamber slide with a chamber depth of 20 μm (Leja Products B.V., Nieuw-Vennep, The Netherlands), and one CASA measurement was performed. Ten fields per sample were examined automatically by the CASA system. The number of spermatozoa analysed was not fixed and varied among samples depending on sperm concentration, as the number of cells included in the analysis was automatically determined by the predefined settings of the CASA software (HTM-IVOS, version 12.3, Hamilton Thorne Biosciences, Beverly, MA, USA). The CASA system was used to determine total motility (TM, %), progressive motility (PM, %), and sperm concentration (spermatozoa per millilitre).

For each animal, the CASA system automatically generated a single representative value for each sperm parameter based on the 10 fields analysed during the measurement. These automatically generated animal-level values were used directly for statistical analysis.

Sperm morphology was assessed using smear preparations obtained from sperm samples. A small volume of sperm was placed near the margin of a microscope slide and mixed with a drop of Eosin. The mixture was then spread across the slide to form a thin smear and allowed to air-dry at room temperature for approximately 2–3 min. After drying, the smears were briefly rinsed with tap water and left to dry again. The prepared slides were examined using an Olympus microscope (BX51, Olympus, Tokyo, Japan). Images were recorded using a digital colour camera (Colour View II, Olympus Tokyo, Japan), and morphometric analyses were carried out with Cell F Imaging Software for Life Science Microscopy (Olympus Soft Imaging Solutions, Münster, Germany). To determine the proportion of spermatozoa with morphological abnormalities, we evaluated 200 sperm cells per sample and expressed the findings as percentages.

Sperm morphological abnormalities were evaluated according to the segment of the spermatozoon affected. Head abnormalities comprised alterations in head shape, whereas neck abnormalities included flexed heads and detached heads. Tail abnormalities included heads without tails, headless tails, broken tails, bent tails, curved tails, coiled tails, and folded tails [53,54].

### 2.4. Histological Assessment

For histological evaluation, testicular tissue samples were processed using a conventional paraffin-embedding protocol (Paraffin Pastilles for Histology, Merck, Germany). The embedded testes were sectioned at 5 µm using a microtome (Cut 4062, Slee, Mainz, Germany). Tissue sections were stained with hematoxylin and eosin (H&E) according to standard histological procedures and examined under a light microscope (Olympus CX41RF, Olympus Life and Material Science, Southend-on-Sea, UK) equipped with an Olympus DP23 digital microscope camera and cellSens Microscope Imaging Software (cellSens ENTRY version 4.3, CS-EN-V4.3-EL, Olympus, Southend-on-Sea, UK).

Histopathological changes were evaluated using two semi-quantitative scoring systems: a modified Cosentino scoring system to grade testicular damage and the Johnsen testicular biopsy scoring system to assess spermatogenesis. Two blinded pathologists performed the grading independently, and the final score for each animal was calculated as the mean of the two observers’ grades.

Testicular histopathological changes were evaluated using a modified scoring system based on the grading approach described by Cosentino et al. [55,56,57]. A single global histopathological score was assigned to each animal by integrating the overall severity of the observed testicular lesions. The original grading approach was adapted to account for the spectrum of histopathological alterations evaluated in the present study. A single global histopathological score was assigned to each animal according to the overall severity and extent of the observed testicular lesions. The assessment included alterations in the organisation of the germinal epithelium and seminiferous tubular architecture, germ-cell and Sertoli-cell vacuolation, germ-cell necrosis, basement membrane detachment, and interstitial oedema. These morphological alterations were evaluated collectively rather than scored separately.

The severity of testicular injury was classified as follows: Grade 0, normal testicular morphology, with preserved seminiferous tubular architecture and orderly organization of the germinal epithelium, without relevant histopathological alterations; Grade 1, mild changes, characterized by limited and slight alterations of the germinal epithelium and/or occasional associated lesions, with overall preservation of seminiferous tubular architecture; Grade 2, moderate changes, characterized by more evident and/or multifocal alterations of the germinal epithelium accompanied by degenerative changes, with partial disruption of normal seminiferous tubular architecture; Grade 3, marked changes, characterized by extensive epithelial disorganization and degenerative lesions, with substantial disruption of seminiferous tubular architecture and/or prominent associated lesions; and Grade 4, severe changes, characterized by widespread and pronounced testicular injury, with extensive loss of normal germinal epithelial and seminiferous tubular architecture and severe degenerative and/or necrotic changes.

The degree of spermatogenesis was further assessed using the Johnsen testicular biopsy scoring system [58] in which seminiferous tubules are assigned a score from 1 to 10 according to the most advanced germ cell type present and the overall organisation of the seminiferous epithelium:

Score 10—complete and normal spermatogenesis with a well-organised germinal epithelium;

Score 9—disorganised germinal epithelium despite the presence of spermatozoa;

Score 8—fewer than 10 spermatozoa in the lumen;

Score 7—numerous spermatids but no spermatozoa;

Score 6—fewer than 10 spermatids without spermatozoa;

Score 5—spermatocytes present but no spermatids or spermatozoa;

Score 4—fewer than five spermatocytes, no other types of germ cells;

Score 3—only spermatogonia present;

Score 2—Sertoli cells present in the absence of germ cells;

Score 1—complete absence of both germ cells and Sertoli cells within the seminiferous tubules.

### 2.5. Statistical Analysis

Data were analysed using R software (version 4.5.2) and presented as mean ± standard deviation (SD). The effects of treatment, generation, and their interactions on sperm parameters and semi-quantitative histopathological scores were analysed using two-way ANOVA followed by Tukey’s honestly significant difference (HSD) post hoc test for multiple comparisons. Pearson’s correlation coefficients were calculated to assess linear trends in sperm parameters across generations within each treated group. Statistical significance was set at *p* > 0.05. To assess the impact of treatment across experimental groups, we calculated percentage changes relative to the corresponding control within each generation using R.

Because more than one male from the same litter could be included in the analyses, we treated individual animals as independent experimental units and did not specifically account for within-litter dependence in the statistical analysis.

The numbers of total pups, alive and dead pups at birth, and alive and dead pups at sexual maturity were analysed using two-way analysis of variance (ANOVA), with generation (F1 and F2) and treatment (Control, E1, E2, and E3) as fixed factors, including their interaction. Because some females did not produce a litter, analyses of litter-related outcomes were performed using females that gave birth (i.e., total pups > 0). When significant differences were detected, Tukey’s honestly significant difference (HSD) test was used for post hoc multiple comparisons. Data are presented as mean ± standard deviation (SD), with *p* < 0.05 considered statistically significant.

## 3. Results

### 3.1. Reproductive Outcomes

Table 1 presents reproductive performance across the F1 and F2 generations. The control group maintained consistently high reproductive performance across all evaluated endpoints and both generations. In the experimental groups, reproductive success generally declined from E1 to E3, with the reduction becoming more apparent at the later stages of the reproductive process. While mating and gestation rates were only moderately affected, parturition showed the clearest decrease, particularly in F2. Accordingly, litter numbers declined across the experimental groups, reaching four in F1–E3 and three in F2–E3, compared with seven in the respective control groups. The lowest parturition rate was recorded in F2–E3 (60%).

As shown in Table 2, offspring viability was progressively reduced across the experimental groups in both the F1 and F2 generations. In the F1 generation, the total number of pups and the number of live pups at birth decreased significantly in the experimental groups compared with the corresponding control, with the most pronounced changes observed in the E3 group. This reduction was accompanied by an increase in the number of dead pups at birth, particularly in E3.

Two-way ANOVA revealed no significant effect of generation on any of the three reproductive parameters (total pups, alive and dead pups at birth, alive and dead pups at sexual maturity), whereas treatment exerted a highly significant effect on all parameters (*p* < 0.001). A significant generation × treatment interaction was observed for the number of alive pups at birth and alive pups at sexual maturity (*p* < 0.05), but not for total pups (*p* > 0.05).

The differences became more evident at sexual maturity, when the number of surviving offspring was significantly lower in all experimental groups compared with the control. Accordingly, postnatal survival declined progressively from E1 to E3, indicating a clear gradient in offspring viability across the experimental groups.

A similar pattern was observed in the F2 generation, although the changes were generally more pronounced than in F1. Significant reductions in total pups and live pups at birth were accompanied by increased mortality. At sexual maturity, the number of surviving offspring declined further, with the greatest reduction again observed in the E3 group. Postnatal survival followed the same decreasing pattern across the experimental groups and was markedly lower in F2 than in F1, particularly in E2 and E3.

Overall, the results indicate a progressive reduction in offspring viability from E1 to E3 in both generations, characterised by decreased survival from birth to sexual maturity and increased offspring mortality. These effects were more pronounced in the F2 generation, with E3 consistently showing the greatest changes (Table 2).

### 3.2. Sperm Assessment

**Figure 2:**
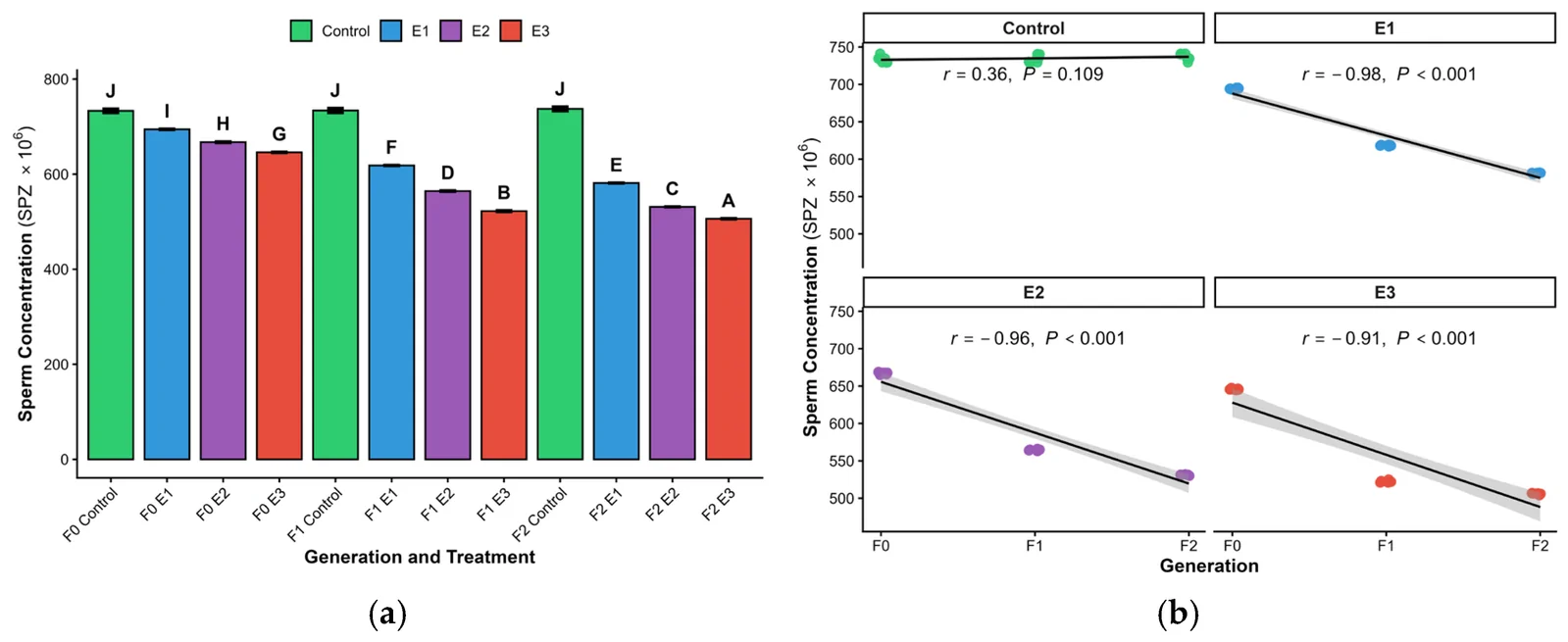
Effect of potassium dichromate on sperm concentration across generations. (**a**) Sperm concentration in control and treated groups across generations. Data are presented as mean ± SD (n = 7 per group). Statistical differences among all experimental groups were analysed using two-way ANOVA followed by Tukey’s HSD multiple-comparison test. Bars with different letters differ significantly (*p* > 0.05), whereas bars sharing at least one letter do not differ significantly. (**b**) Pearson correlation analysis between generation and sperm concentration. Each dot represents an individual animal (n = 7 per group). All treated groups showed a significant negative correlation (*p* > 0.0001), while the control group showed no significant correlation.

Two-way ANOVA revealed significant effects of generation, treatment and their interaction in all evaluated sperm parameters (all *p* > 0.0001). Tukey’s HSD post hoc analysis demonstrated a significant decrease in sperm concentration in all treated groups compared with their corresponding control group, *p* > 0.05 (Figure 2a).

However, sperm concentration decreased progressively from E1 to E3 within each generation, highlighting a dose-dependent negative effect of potassium dichromate on sperm concentration. Furthermore, the reduction was more pronounced across generations, indicating a cumulative effect. Relative to the corresponding control group within each generation, the reduction increased from approximately 5.3–11.9% in F0 to 21.14–31.35% in F2.

Pearson’s correlation analysis confirmed a significant, strong negative association between generations and sperm concentration within each treated group (*p* < 0.0001), indicating a progressive decline in sperm concentration from F0 to F2, while the control group showed no significant correlation (Figure 2b).

**Figure 3:**
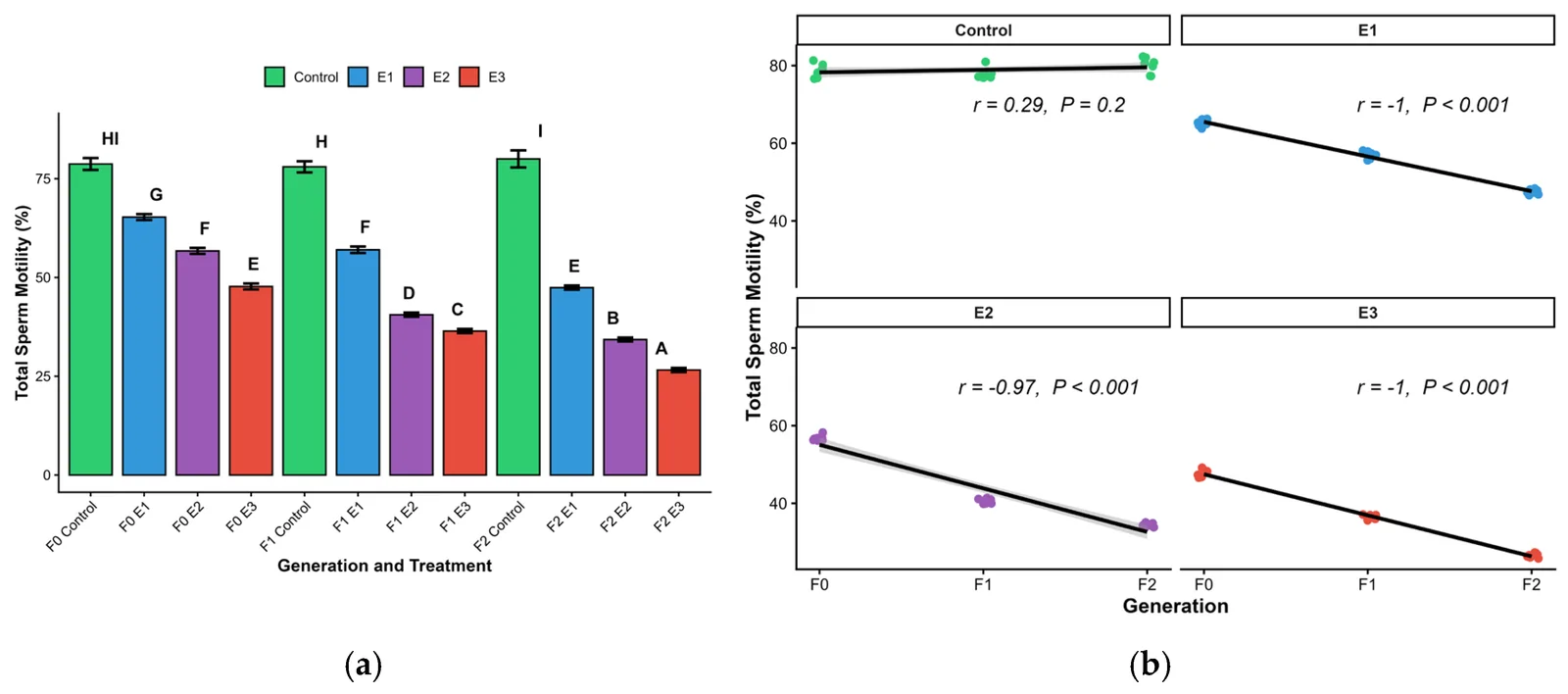
Effect of potassium dichromate on total sperm motility across generations. (**a**) Total sperm motility in control and treated groups across generations. Data are presented as mean ± SD. (n = 7 per group). Statistical differences among all experimental groups were analysed using two-way ANOVA followed by Tukey’s HSD multiple-comparison test. Bars with different letters differ significantly (*p* > 0.05), whereas bars sharing at least one letter do not differ significantly. (**b**) Pearson correlation analysis between generation and total sperm motility. Each dot represents an individual animal (n = 7 per group). All treated groups showed a significant negative correlation (*p* > 0.0001), while the control group showed no significant correlation.

Regarding total sperm motility, Tukey’s HSD post hoc analysis showed a significant reduction in all treated groups compared with their corresponding controls (*p* > 0.05) (Figure 3a). Within each generation, increasing chromium exposure levels were associated with a significant decrease in total sperm motility from E1 to E3, indicating a dose-dependent adverse effect of Cr(VI).

Furthermore, the reduction became more pronounced across F0 to F2, suggesting a cumulative effect of Cr(VI). Specifically, compared with the corresponding control group within each generation, total sperm motility was reduced by 17–39.38% in F0 and by 40.7–66.79% in F2.

Total sperm motility was also significantly affected by generation (Figure 3b), showing a progressive decline from F0 to F2 across all exposure groups. Pearson’s correlation analysis revealed a significant negative correlation between generation and total sperm motility (*p* > 0.0001), irrespective of Cr(VI) exposure level. No significant correlation was observed in the control group.

**Figure 4:**
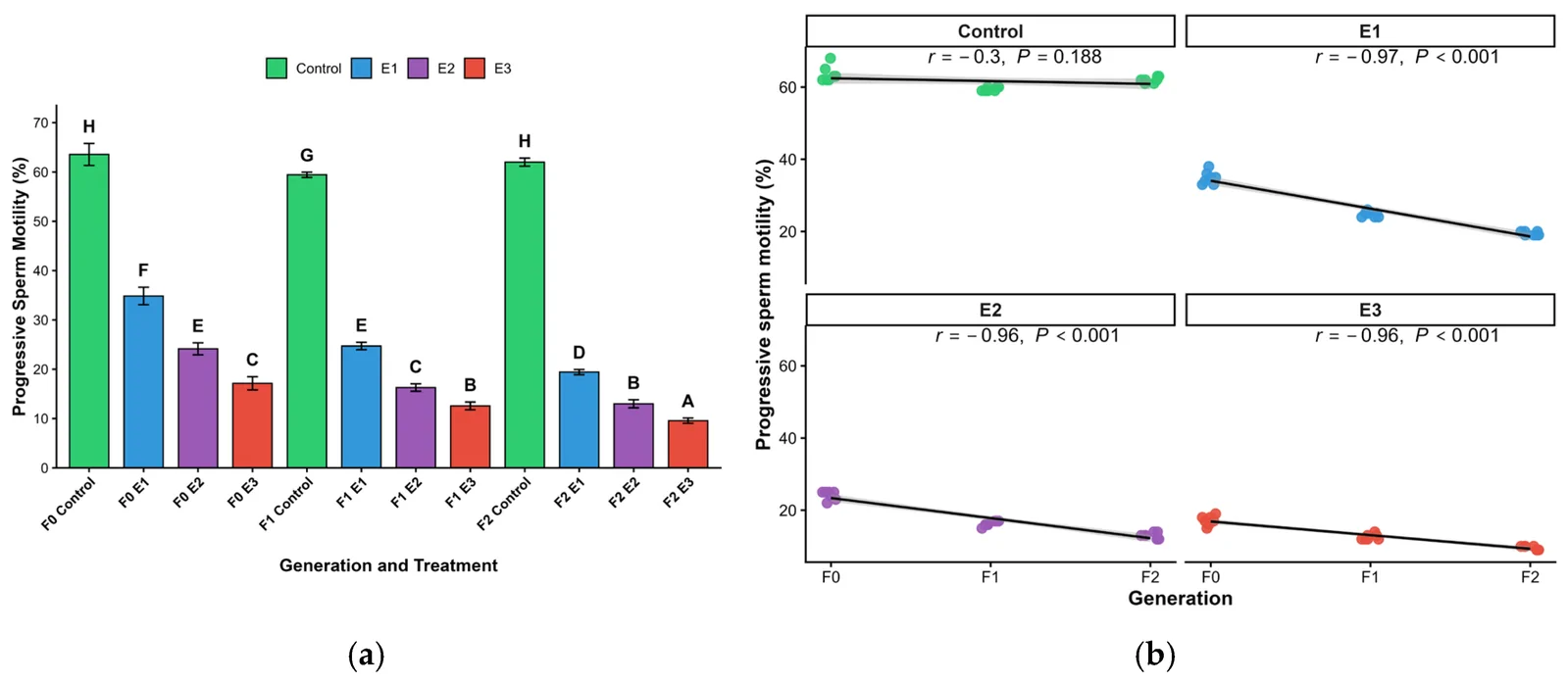
Effect of potassium dichromate on progressive sperm motility across generations. (**a**) Progressive sperm motility in control and treated groups across generations. Data are presented as mean ± SD (n = 7 per group). Statistical differences among all experimental groups were analyzed using two-way ANOVA followed by Tukey’s HSD multiple-comparison test. Bars with different letters differ significantly (*p* > 0.05), whereas bars sharing at least one letter are not significantly different. (**b**) Pearson correlation analysis between generation and progressive sperm motility. Each dot represents an individual animal (n = 7 per group). A significant negative correlation was observed in all treated groups (*p* > 0.0001), while the control group showed no significant correlation.

Progressive sperm motility (Figure 4a) followed the same pattern as sperm concentration and total sperm motility, showing a significant (*p* > 0.05) decrease in all groups exposed to hexavalent chromium in comparison to the corresponding control, showing a dose-dependent effect. Moreover, the reduction became progressively more pronounced across successive generations. Specifically, relative to the corresponding control group within each generation, progressive sperm motility was reduced by 45–73% in F0 and by 68.6–84.5% in F2.

Pearson’s correlation analysis demonstrated a significant negative correlation between generation and progressive sperm motility in all treated groups (*p* > 0.0001), whereas the control group showed no significant correlation (Figure 4b).

**Figure 5:**
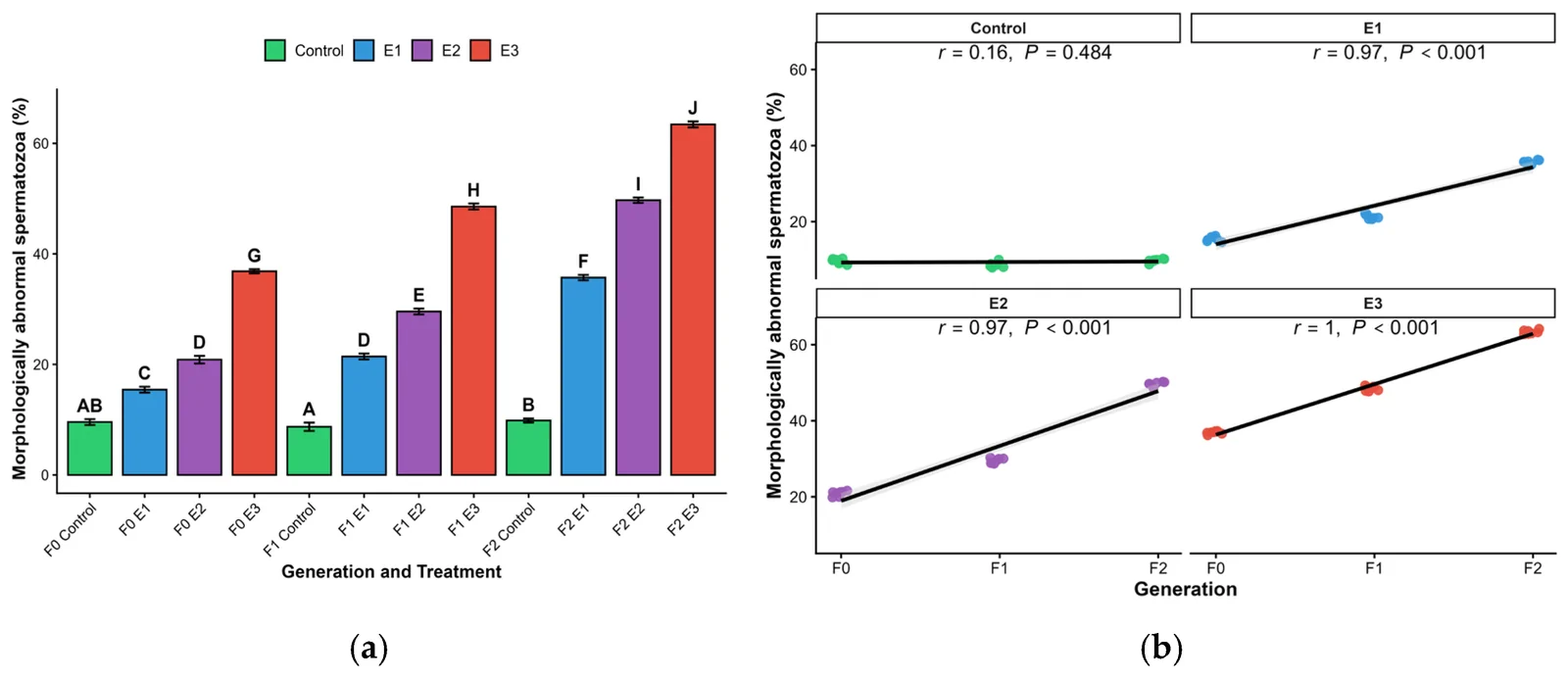
Effect of potassium dichromate on the percentage of morphologically abnormal spermatozoa across generations. (**a**) Percentage of abnormal sperm in control and treated groups across generations. Data are presented as mean ± SD (n = 7 per group). Statistical differences among all experimental groups were analysed using two-way ANOVA followed by Tukey’s HSD multiple-comparison test. Bars with different letters differ significantly (*p* > 0.05), whereas bars sharing at least one letter do not differ significantly. (**b**) Pearson correlation analysis between generation and morphologically abnormal spermatozoa. Each dot represents an individual animal (n = 7 per group). All treated groups showed a significant positive correlation (*p* > 0.0001), while the control group showed no significant correlation.

The percentage of morphologically abnormal spermatozoa increased significantly in a dose-dependent manner in all Cr(VI)-treated groups compared with the corresponding control group within each generation (*p* < 0.05; Figure 5a). Moreover, the magnitude of this increase became more pronounced across successive generations, ranging from 61.2–285.1% in F0, corresponding to actual values of 15.43–36.86% compared with 9.57% in the F0 control group, to 262.3–543.4% in F2, corresponding to actual values of 35.71–63.43% compared with 9.86% in the F2 control group.

Additionally, Pearson’s correlation analysis showed a significant positive correlation between generation and the percentage of morphologically abnormal spermatozoa in all treated groups (*p* > 0.0001), whereas the control group showed no significant correlation (Figure 5b).

**Figure 6:**
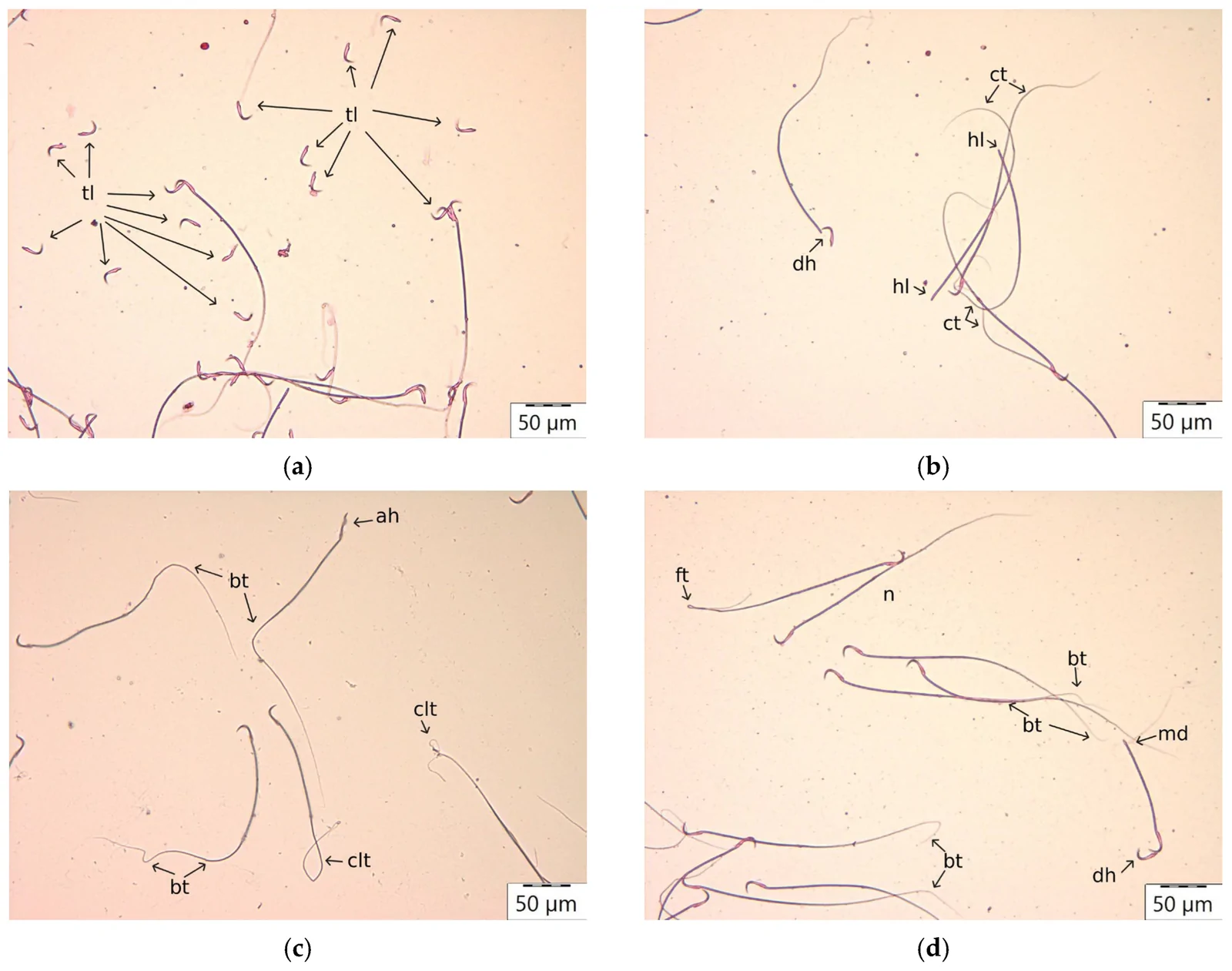
Spermatozoa with morphological anomalies stained with Eosin, ob. 20X. (**a**) tailless—tl; (**b**) curved tail—ct, headless—hl, detached head—dh; (**c**) bent tail—bt, coiled tail—clt, amorphous head—ah; (**d**) normal—n, bent tail—bt, folded tail—ft, detached head—dh, membrane damage—md.

Sperm abnormalities observed in chromium-exposed animals were evaluated based on the region of the spermatozoon affected (Figure 6). Across all generations, chromium exposure induced abnormalities affecting the head, neck, and tail regions. Head abnormalities comprised alterations in head shape, including amorphous heads, whereas neck abnormalities included flexed heads and detached heads. Tail abnormalities included heads without tails, headless tails, broken tails, bent tails, curved tails (involving the midpiece and/or distal part of the tail), coiled tails, and folded tails. Membrane damage was also observed in spermatozoa from chromium-exposed animals.

Among the observed morphological defects, tail abnormalities appeared to predominate in chromium-exposed animals.

### 3.3. Histological Assessment

**Figure 7:**
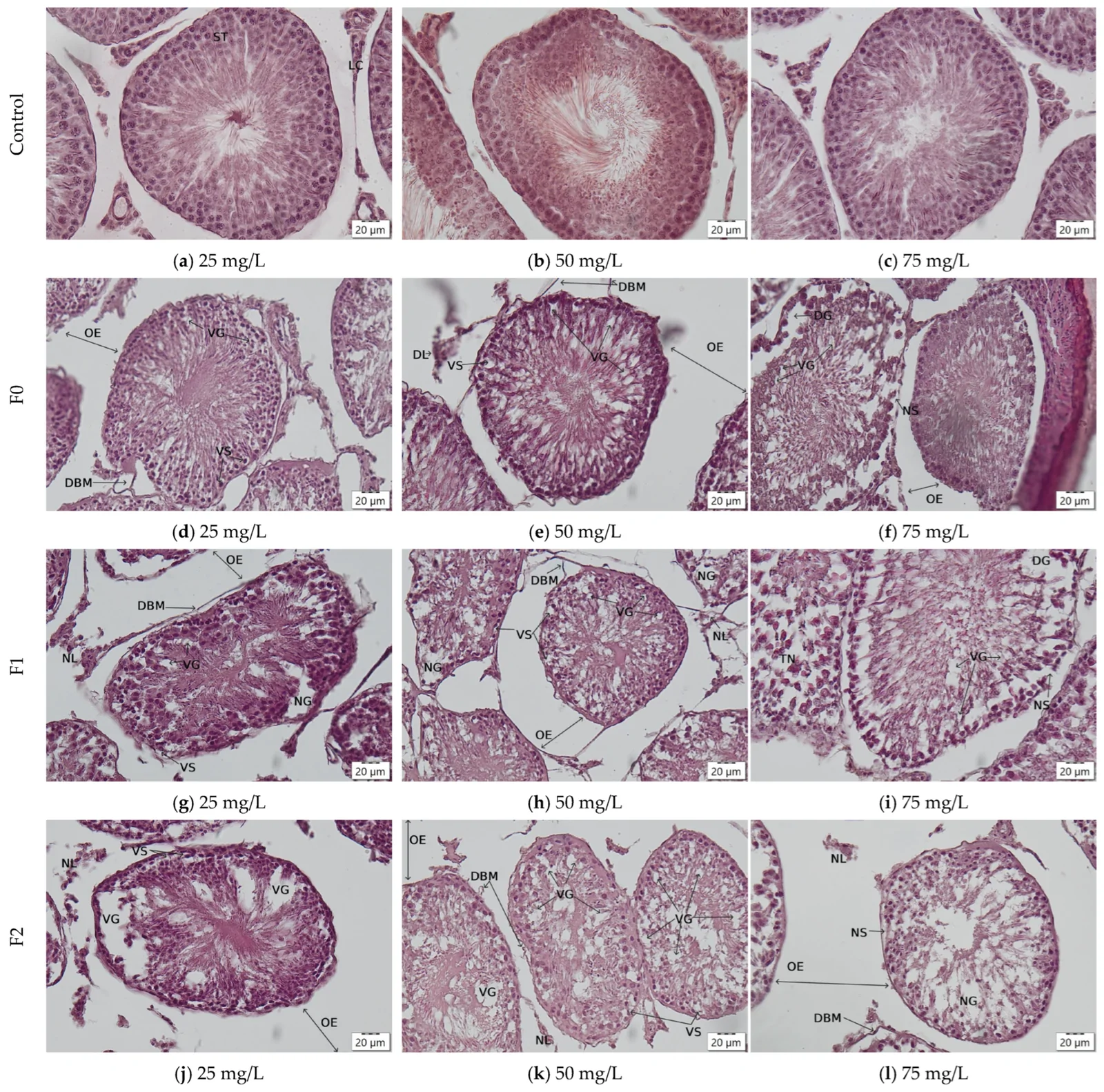
Histological architecture of the testes across three generations, H.E. stain, ob. 40X. OE—oedema, DBM—detachment of the seminiferous tubular basement membrane, VG—vacuolation of germinal epithelium, DL—degeneration of Leydig cells, VS—vacuolation of Sertoli cells, DG—detachment of germinal epithelium from Sertoli cells, NS—necrosis of Sertoli cells, NG—necrosis of germinal cells, NL—necrosis of Leydig cells, TN—tubular necrosis with total disorganization of germinal epithelium marked by necrosis of germinal and Sertoli cells, and absence of basement membrane.

Histopathological alterations of the testicular architecture (Figure 7) were observed in all potassium dichromate-exposed groups across the three generations and were characterised by lesions consistent with degenerative changes. Multifocal degeneration of the seminiferous epithelium was evidenced by disorganisation of the germinal epithelium with an apparent reduction in germ cell density and disruption of the normal cellular architecture. Detached germ cells were present within the seminiferous tubule lumen. Vacuolation of the seminiferous epithelium, affecting both Sertoli cells and germ cells, was common. Focal detachment or absence of the seminiferous tubular basement membrane was also observed. Together with Sertoli cell degeneration, these lesions may compromise the integrity of the blood–testis barrier, thereby contributing to impaired spermatogenesis.

The interstitial compartment was likewise affected. Vacuolar degeneration of Leydig cells, frequently accompanied by necrosis, was observed in the exposed groups. Retention of mature spermatozoa within the Sertoli cell layer was identified in some seminiferous tubules, indicating defective spermiation.

**Figure 8:**
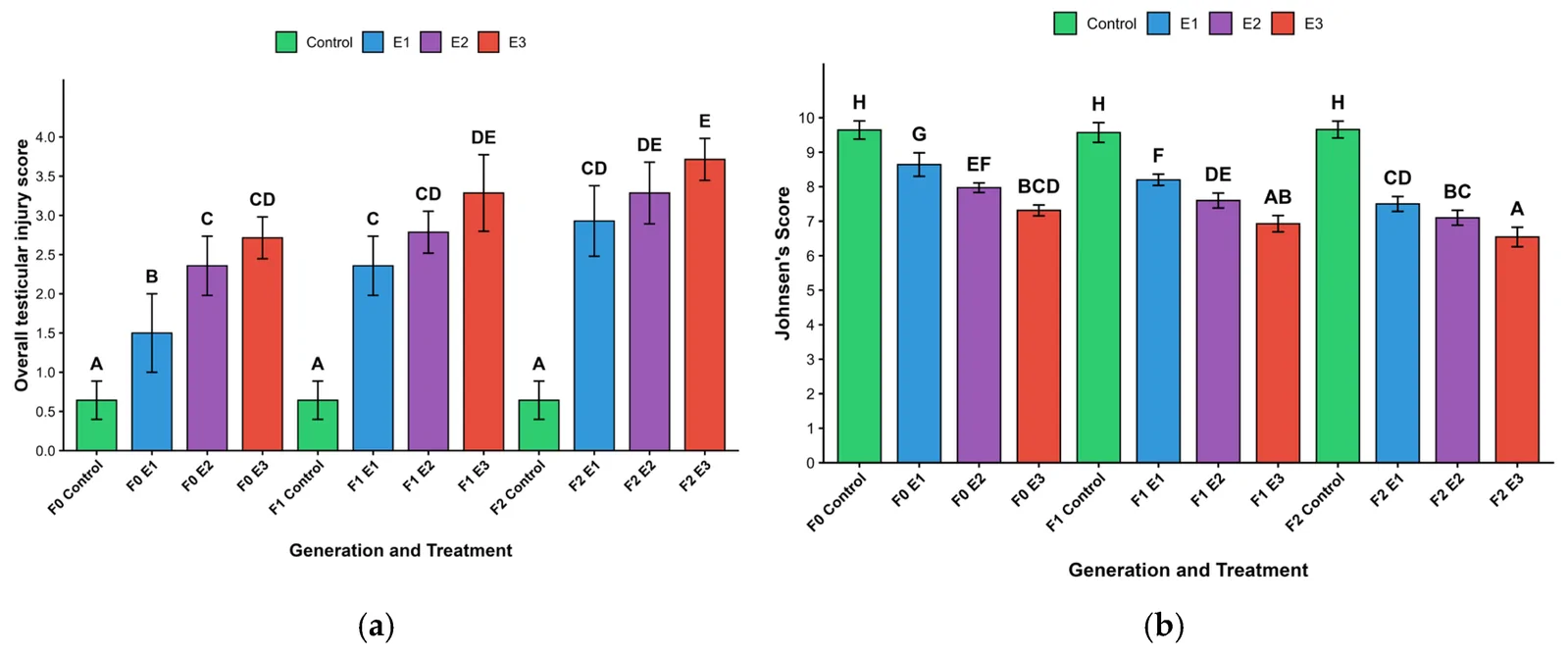
Semi-quantitative histopathological assessment of testicular lesions following potassium dichromate exposure across three generations. (**a**) Overall testicular injury score. (**b**) Johnsen’s spermatogenesis score. Data are presented as mean ± SD (n = 7 animals per group). Statistical differences among all experimental groups were analyzed using two-way ANOVA followed by Tukey’s HSD multiple-comparison test. Bars sharing at least one letter are not significantly different, whereas bars with no common letters differ significantly (*p* < 0.05).

Two-way ANOVA revealed significant effects of generation, treatment, and their interaction on all evaluated histopathological scores (*p* > 0.0001). The overall severity of testicular histopathological injury was further assessed using the modified semi-quantitative Cosentino scoring system (Figure 8a). The score increased progressively with increasing chromium dose within each generation, demonstrating a dose-dependent adverse effect. Additionally, lesion severity became more pronounced across successive generations, suggesting a cumulative effect. Notably, mean injury scores increased from 1.50–2.71 in the F0 to 2.93–3.71 in the F2 generation compared to the corresponding control group within each generation, with the highest score in the F2–E3 group.

The degree of spermatogenesis was further evaluated using the Johnsen scoring system (Figure 8b). Consistent with the histopathological alterations of the seminiferous epithelium, the degeneration of Sertoli and Leydig cells, a progressive decline in Johnsen scores was observed with increasing potassium dichromate exposure within each generation, demonstrating a dose-dependent impairment of spermatogenesis. Furthermore, the reduction became more pronounced across successive generations, suggesting a cumulative effect. Mean Johnsen scores declined from 8.64–7.31 in the F0 to 7.5–6.54 in the F2 generation compared with the corresponding control group within each generation, with the lowest score recorded in the F2–E3 generation.

## 4. Discussion

The results showed a decline in sperm quality across all exposed groups, proportional to the administered potassium dichromate dose and the number of generations analysed. Sperm quality was assessed based on several biological markers, including sperm concentration, total motility, progressive motility, and the percentage of abnormal spermatozoa. Sperm quality was significantly impaired following exposure to hexavalent chromium, with the highest concentration causing the most severe effects. Compared with the control group, sperm concentration, total motility, and progressive motility decreased, while the percentage of sperm abnormalities increased, and this trend intensified with each successive generation. These findings are consistent with our previous study, which demonstrated dose-dependent chromium accumulation in the reproductive organs of both F0 and F1 animals, with higher chromium levels detected in the F1 generation, suggesting greater susceptibility of the developing reproductive system to chromium toxicity [47].

Impaired sperm quality was accompanied by declines in reproductive performance and offspring viability. Although mating was largely maintained, gestation and parturition success, as well as litter number, declined with increasing potassium dichromate exposure, with more pronounced effects in the F2 generation. This pattern was accompanied by reduced litter size and offspring survival and increased neonatal mortality. Taken together, these findings suggest that the progressive deterioration of male reproductive parameters was associated with reduced reproductive success and compromised offspring viability, particularly at higher exposure levels and after repeated generational exposure.

Although various experimental models have demonstrated the negative effects of hexavalent chromium on male reproductive function, this study’s novelty lies in its multigenerational toxicity analysis. The current scientific literature does not report studies extending beyond the parental and F1 generations. Given the well-documented oxidative and reproductive-disrupting nature of hexavalent chromium, this study aimed to investigate the effects of long-term exposure. This research is particularly relevant considering that numerous industrial sources of hexavalent chromium pollution are located near areas inhabited by humans and animals.

Following oral exposure, only a fraction of ingested Cr(VI) reaches the systemic circulation, as a substantial proportion is reduced to Cr(III) in the acidic environment of the stomach. The two chromium species differ markedly in their cellular uptake mechanisms. Cr(III) has limited ability to cross cellular membranes and may enter cells through pinocytosis or other nonspecific mechanisms, whereas Cr(VI), owing to its structural similarity to physiological anions, can enter cells through anion transport systems [59]. This greater cellular permeability contributes to the substantially higher toxicity of Cr(VI). Once absorbed, chromium may exert toxic effects locally or be distributed to distant tissues and organs [59]. In the context of the present study, systemic distribution following gastrointestinal absorption provides a plausible route by which potassium dichromate-derived chromium may reach the testes and contribute to observed testicular alterations.

Previous studies have demonstrated that hexavalent chromium crosses the placental barrier, accumulates in fetal tissues including the testes, and disrupts testicular development and steroidogenesis in the offspring [11,46,50,60,61]. In addition, chromium can be transmitted through maternal milk, thereby contributing to postnatal exposure during lactation [62]. Consistent with this mechanism, the F1 and F2 offspring in our study were exposed prenatally via placental transfer, postnatally through lactation, and, after weaning, through potassium dichromate administered in drinking water according to the experimental protocol. Such exposure during critical periods of development may also have contributed to the reduced offspring viability and postnatal survival observed in the exposed groups.

Hexavalent chromium is recognised as an environmental endocrine disruptor that interferes with the hypothalamic–pituitary–gonadal (HPG) axis, leading to reproductive dysfunction [63]. Within this axis, LH stimulates Leydig cells to produce testosterone, which is essential for the maintenance of spermatogenesis through its action on Sertoli cells [9]. Therefore, disruption of LH secretion or Leydig cell function by chromium exposure may impair testosterone production, ultimately compromising spermatogenesis and sperm quality.

Our earlier multigenerational investigations (F0–F2) also confirmed the endocrine toxicity of hexavalent chromium, revealing disrupted reproductive hormone homeostasis characterised by reduced circulating testosterone and elevated LH levels compared with control animals. Moreover, these endocrine disturbances became increasingly severe with both higher chromium doses and repeated generational exposure, resulting in a progressive reduction in circulating testosterone and a corresponding elevation in LH concentrations, a pattern consistent with chronic dysregulation of the HPG axis [48]. The endocrine disturbances induced by chromium exposure may represent a key link to the impaired sperm quality observed in the present study, with the most severe effects occurring in the F2 generation, consistent with the poorer reproductive performance and offspring viability observed in this generation.

The endocrine alterations described above are likely explained, at least in part, by chromium accumulation within the testes. Chromium accumulates in the testes [64], where it induces structural alterations, particularly in Leydig cells, and suppresses the expression of key steroidogenic enzymes involved in androgen biosynthesis [11,38]. These findings are consistent with the endocrine alterations observed in our previous multigenerational [48] studies and provide a mechanistic basis for the histopathological lesions identified in the present investigation.

Sertoli cells are essential for maintaining spermatogenesis and the integrity of the blood–testis barrier [11,62,65]. Kumar et al. [46] demonstrated that in utero exposure to hexavalent chromium induced structural and functional alterations in Sertoli and germ cells of F1 offspring, suggesting that disruption of Sertoli cell function and blood–testis barrier integrity contributes to impaired spermatogenesis.

Subramanian et al. also demonstrated disruption of testicular histoarchitecture—and, consequently, testicular function. Ref. [37], who investigated the effects of hexavalent chromium in monkeys. Furthermore, exposure of pregnant female rats to chromium has been shown to cause similar alterations in the testicular structure and function of F1 offspring, due to the transfer of chromium both through the placenta and via maternal milk [46].

Consistent with these mechanisms, histopathological examination in our study revealed significant, dose-dependent testicular lesions, including degeneration and necrosis of the seminiferous epithelium, interstitial oedema, and marked impairment of Leydig and Sertoli cells. The widespread disorganisation of the seminiferous epithelium, associated with depletion of germ cells, detachment of immature germ cells into the tubular lumen, and epithelial vacuolation, indicates a profound disruption of the spermatogenic compartment. In this context, Cesta et al. [66] described seminiferous epithelial vacuolation as a feature associated with tubular degeneration or atrophy, with vacuoles representing spaces resulting from the loss of cells from the seminiferous epithelium. Thus, the vacuolar changes observed in the present study further support the progressive degeneration of the seminiferous tubules and are consistent with the reduced Johnsen scores in exposed animals, reflecting a gradual decline in spermatogenic efficiency with increasing chromium exposure. Furthermore, the observed Sertoli cell alterations, together with focal disruption of the seminiferous tubular basement membrane, may indicate compromised structural integrity of the spermatogenic microenvironment. Given the essential structural, nutritional, and regulatory roles of Sertoli cells in germ cell development, such alterations may contribute to impaired germ cell differentiation and spermatogenesis.

The interstitial compartment was similarly affected, with vacuolar degeneration and Leydig cell necrosis suggesting impaired steroidogenic function. This interpretation is supported by our previous study [48], in which reduced testosterone concentrations were observed in exposed animals. Although testosterone was not measured in the present study, the Leydig cell alterations identified here, considered together with our previously reported hormonal findings [48], may indicate disruption of testicular endocrine function. As described by Cesta et al. [66], impaired testosterone production can interfere with spermiation, leading to sperm retention and potentially contributing to adverse changes in sperm concentration and morphology. Thus, the Leydig cell alterations observed in the present study may contribute to the deterioration of sperm concentration and morphology following potassium dichromate exposure. In parallel, the progressive increase in interstitial oedema may reflect vascular and inflammatory alterations that could further aggravate testicular damage by disturbing tissue homeostasis and the microenvironment required for normal spermatogenesis.

Importantly, the semiquantitative histopathological assessment demonstrated a clear progression in lesion severity across successive generations, as reflected by increasing Cosentino scores and declining Johnsen scores. The predominance of extensive degeneration and coagulative necrosis of the seminiferous epithelium in the F2 generation indicates that repeated multigenerational exposure results in cumulative structural damage to the testes. Collectively, these findings provide compelling morphological evidence that chronic potassium dichromate exposure progressively compromises both the exocrine and endocrine functions of the testis.

Oxidative stress is considered one of the principal mechanisms underlying chromium-induced reproductive toxicity [18,62] and has been associated with histological damage, including degeneration of the seminiferous tubule epithelium and disruption of spermatogenesis [67]. Previous studies have shown that excessive ROS production can promote lipid peroxidation, germ cell apoptosis, and mitochondrial dysfunction, potentially contributing to impaired spermatogenesis and reduced sperm concentration and motility [37,46]. This vulnerability has been attributed to the high content of polyunsaturated fatty acids in the sperm cell membrane, which makes it susceptible to the damaging effects of ROS [39]. Protein phosphorylation within the sperm membrane may also be affected by hydrogen peroxide and hydroxyl radicals, potentially compromising the structural integrity of the flagellum and reducing sperm motility [68]. Furthermore, Cr(VI)-induced mitochondrial dysfunction has been proposed to impair ATP production and, consequently, the energy supply required for sperm motility [69]. These mechanisms, although supported by previous studies, were not directly assessed in the present study and should therefore be considered potential explanations for the observed reproductive effects.

The increase in the percentage of spermatozoa with abnormalities is closely correlated with both the level and duration of exposure to hexavalent chromium, with the most severe effects observed in individuals from the F2 generation. Based on previous studies, these abnormalities may be associated with chromium-induced oxidative stress [25], and impaired Sertoli cells function [11,70], which could contribute to alterations in spermatogenesis and sperm maturation.

Sperm abnormalities observed following Cr(VI) exposure affected different regions of the spermatozoon. Head abnormalities included changes in head shape, such as amorphous heads, while neck abnormalities included flexed and detached heads. Heads without tails were also observed. Previous studies have associated these morphological alterations with disturbances in spermiogenesis, chromatin condensation, and DNA integrity [70]. Tail abnormalities included broken, bent, curved, coiled, and folded tails. Abnormalities involving the midpiece may be associated with chromium-induced mitochondrial dysfunction [69], whereas alterations of the tail may reflect cytoskeletal disruption and impaired Sertoli cell support. These morphological changes could potentially be associated with Cr(VI)-induced apoptotic or degenerative processes in Sertoli cells mediated by ROS generation [11,70]. However, because the present study did not directly assess mitochondrial damage and apoptosis, these mechanisms remain possible explanations for the observed morphological alterations.

The concentrations used in the present study (25–75 mg/L K_2_Cr_2_O_7_) should be regarded as controlled experimental exposures designed to characterise concentration-related reproductive toxicity rather than as direct equivalents of human occupational or environmental exposure. Nevertheless, the biological relevance of the lower concentration is supported by previous experimental studies reporting toxicological and reproductive effects at comparable exposure levels [49,50]. Differences in exposure route, duration, chromium speciation, and toxicokinetics should therefore be considered when extrapolating these findings to humans.

Several limitations of the present study should be acknowledged. Although the findings indicate concentration- and generation-dependent adverse effects of hexavalent chromium on male reproductive parameters, the experimental design does not allow conclusions regarding true transgenerational inheritance, as the F1 and F2 generations were directly and/or developmentally exposed to the toxicant. In addition, because more than one male from the same litter were included in the analyses, within-litter dependence was not specifically accounted for statistically, and a potential influence of litter effects on the observed outcomes cannot be excluded. Furthermore, although the observed functional and histopathological alterations are consistent with oxidative stress-mediated reproductive toxicity, the molecular pathways underlying these changes were not investigated. Further studies incorporating litter as a statistical factor, together with molecular and epigenetic analyses and experimental designs including unexposed subsequent generations, are needed to further clarify the mechanisms underlying the persistence of chromium-induced reproductive toxicity.

## 5. Conclusions

This study demonstrates that hexavalent chromium exposure induces dose-dependent and generation-dependent impairment of male reproductive function, as shown by the progressive decline in sperm quality and associated histopathological alterations in the testes. Animals from subsequent generations, particularly the F2 generation, exhibited more pronounced changes in sperm concentration, motility, morphology, and testicular architecture than those from earlier generations. These findings indicate that continuous exposure to hexavalent chromium across successive generations is associated with cumulative adverse effects on male reproductive health. Overall, the results provide experimental evidence that prolonged chromium exposure progressively impairs spermatogenesis and testicular integrity, highlighting the reproductive risks associated with chronic environmental exposure to this metal.

## Figures and Tables

**Table 1:** Reproductive performance in the F1 and F2 generations.

Generation	Group	Mated Pairs (Male/Female)	Mating Rate n/N (%)	Gestation Rate n/N (%)	Parturition Rate n/N (%)	Number of Litters
F1	C	7/7	7/7 (100%)	7/7 (100%)	7/7 (100%)	7
	E1	7/7	7/7 (100%)	6/7 (85.7%)	6/6 (100%)	6
	E2	7/7	7/7 (100%)	6/7 (85.7%)	5/6 (83.33%)	5
	E3	7/7	6/7 (85.7%)	5/6 (83.33%)	4/5 (80%)	4
F2	C	7/7	7/7 (100%)	7/7 (100%)	7/7 (100%)	7
	E1	7/7	7/7 (100%)	6/7 (85.7%)	5/6 (83.33%)	5
	E2	7/7	6/7 (85.7%)	5/6 (83.33%)	4/5 (80%)	4
	E3	7/7	6/7 (85.7%)	5/6 (83.33%)	3/5 (60%)	3

**Table 2:** Offspring viability in the F1 and F2 generations.

Generation	Group	Total Number of Pups at Birth (Mean ± SD)	Alive Pups at Birth (Mean ± SD)	Dead Pups at Birth (Mean ± SD)	Alive Pups at Sexual Maturity (Mean ± SD)	Dead Pups at Sexual Maturity (Mean ± SD)	Postnatal Survival Rate (%)
F1	C	10.43 ± 0.98	10.43 ± 0.98	0.00 ± 0.00	10.43 ± 0.98	0.00 ± 0.00	100
	E1	9.17 ± 0.41 *	8.67 ± 0.52 ***	0.50 ± 0.55 ns	8.17 ± 0.75 ***	0.50 ± 0.55 ns	94.23
	E2	8.60 ± 0.55 **	7.80 ± 0.45 ***	0.80 ± 0.45 *	7.00 ± 0.71 ***	0.80 ± 0.45 ns	89.74
	E3	8.25 ± 0.96 ***	6.75 ± 0.50 ***	1.50 ± 0.58 ***	5.25 ± 0.96 ***	1.25 ± 0.50 *	77.77
F2	C	10.86 ± 0.69	10.86 ± 0.69	0.00 ± 0.00	10.86 ± 0.69	0.00 ± 0.00	100
	E1	8.80 ± 0.45 ***	8.00 ± 0.00 ***	0.80 ± 0.45 *	7.20 ± 0.84 ***	0.80 ± 0.84 ns	90
	E2	8.25 ± 0.50 ***	7.00 ± 0.82 ***	1.25 ± 0.50 ***	5.75 ± 0.50 ***	1.25 ± 0.96 *	82.14
	E3	7.67 ± 0.58 ***	5.67 ± 0.58 ***	2.00 ± 0.00 ***	3.67 ± 1.15 ***	2.00 ± 1.00 ***	64.7

Statistical significance was indicated for treatment groups compared with the corresponding control group within the same generation. The statistical significance was indicated as follows: *p* < 0.05 (*), *p* < 0.01 (**), and *p* < 0.001 (***). Values with *p* ≥ 0.05 were considered non-significant (ns).

## Data Availability

The original contributions presented in this study are included in the article. Further inquiries can be directed to the corresponding authors.

## Abbreviations

The following abbreviations are used in this manuscript:

C	Control group
E	Experimental group
CASA	computer-assisted sperm analysis
ROS	reactive oxygen species
Cr(VI)	hexavalent chromium
Cr(III)	trivalent chromium
HPG	hypothalamic-pituitary-gonadal
LH	luteinizing hormone
